# Linear and curvilinear associations between affinity for solitude and adjustment in adolescence

**DOI:** 10.1111/jora.70129

**Published:** 2025-12-29

**Authors:** Jiaxi Zhou, Xinyin Chen, Dan Li, Junsheng Liu, Tong Zhou

**Affiliations:** ^1^ Graduate School of Education University of Pennsylvania Philadelphia PA USA; ^2^ School of Psychology Shanghai Normal University Shanghai China; ^3^ School of Psychology and Cognitive Science East China Normal University Shanghai China

**Keywords:** adolescents, affinity for solitude, social, school, and psychological adjustment

## Abstract

This 1‐year longitudinal study examined the linear and nonlinear associations between affinity for solitude and social, school, and psychological adjustment in adolescents. Participants included 2675 eighth‐grade students (1341 boys; *M*_age_ = 14.3 years) in China. Affinity for solitude was assessed through self‐reports, and adjustment data were collected from multiple sources. Results showed that affinity for solitude was positively associated with later prosociality. Moreover, affinity for solitude at low to moderate levels, but not at moderate to high levels, positively predicted leadership. Affinity for solitude at low to moderate levels was negatively associated with later aggression, behavioral problems, and learning problems, but the associations were nonsignificant at moderate levels and positive at higher levels. In addition, affinity for solitude at higher levels, but not at low to moderate levels, negatively predicted school attitudes. The results suggest that the moderate level of affinity for solitude is most beneficial for adolescents' adjustment.

As a salient developmental phenomenon, from late childhood to adolescence, individuals are increasingly inclined to spend time alone (e.g., Coplan et al., 2019; Larson & Richards, 1991). It has been argued that as vigilance to social evaluations by others becomes heightened in adolescence, the experience of solitude may help reduce the pressure of fulfilling social demands and alleviate concerns about conforming to peers (Nguyen et al., 2018; Somerville, 2013). Research has shown that compared with their younger counterparts, adolescents report more positive affect toward solitude and express greater satisfaction about solitary activities (e.g., Danneel et al., 2018; Majorano et al., 2015; Teppers et al., 2013). Enjoyment of spending time alone, referred to as affinity for solitude, as one of the major motivations for solitude in adolescents, and its developmental significance for social, academic, and psychological adjustment have become an important issue in the field (e.g., Borg & Willoughby, 2022; Chen, Zhou, Li, Liu, & Cui, 2024; Goossens, 2013).

Affinity for solitude shares conceptual overlap with the constructs of preference for solitude (Burger, 1995), unsociability (Coplan & Weeks, 2010), introversion (Zelenski et al., 2014), and intrinsically motivated solitude (Nguyen et al., 2018), but it is distinct from the other constructs in its explicit focus on the positive emotional experience of time spent alone (e.g., Chen, Zhou, Li, Liu, & Cui, 2024; Daly & Willoughby, 2020; Hu et al., 2022). For example, preference for solitude and unsociability describe a behavioral tendency to choose spending time alone rather than being with others because social engagement is perceived as uninteresting or unnecessary (Coplan et al., 2019; Coplan & Weeks, 2010). Individuals who prefer solitude over being with others or are unsociable may not necessarily enjoy solitude (Daly & Willoughby, 2020). Introversion is a personality trait characterized by its focus on the internal world and low reward value of social interaction, and introverts do not necessarily seek or enjoy solitude (Zelenski et al., 2014). Affinity for solitude is defined as the experience of positive feelings (i.e., enjoyment, satisfaction) about spending time alone, with a clear feature of affective involvement (Nguyen et al., 2022). The main features of affinity for solitude and related constructs are summarized in Table S1.

It has been argued that affinity for solitude may play a significant role in adolescents' social, academic, and psychological adjustment (e.g., Borg & Willoughby, 2022; Chen, Zhou, Li, Liu, & Cui, 2024). Empirically, the results of research on the relations between affinity for solitude and adolescent adjustment have been mixed. Whereas some studies indicated that affinity for solitude was associated with negative adjustment outcomes, such as depression and peer problems (e.g., Borg & Willoughby, 2022, 2023), other studies revealed that affinity for solitude might promote positive adjustment by fostering restoration, autonomy, and a sense of competence in completing independent tasks (e.g., Chen, Zhou, Li, Liu, & Cui, 2024; Nguyen et al., 2022). There are also studies showing that affinity for solitude was not significantly associated with adjustment outcomes (e.g., Daly & Willoughby, 2020). These results suggest that affinity for solitude may be associated with adjustment outcomes in a complicated manner and further investigation is clearly needed on this issue. Therefore, we conducted the present 1‐year longitudinal study in a sample of Chinese adolescents to examine linear as well as curvilinear associations between affinity for solitude and social, academic, and psychological adjustment.

## AFFINITY FOR SOLITUDE AND ADJUSTMENT IN ADOLESCENCE

The developmental theories (e.g., Kagitcibasi, 2005; Ryan & Deci, 2000) emphasize the inherent needs of human beings for independence or autonomy and social connectedness and their roles in adaptive development. Affinity for solitude may be associated with these needs and their functions. On the one hand, strong affinity for solitude may jeopardize adolescents' connectedness with their peers, failing to meet their need for social belonging. Solitude‐seeking behavior can contribute to social isolation, which is a potential risk factor for adolescents' development because it may limit opportunities to learn necessary social skills, such as cooperation and problem‐solving, and to obtain support from others for performing on school tasks and coping with challenges. The lack of peer connectedness, in turn, may contribute to school and psychological challenges, including negative school attitudes and reduced academic motivation (Nelson et al., 2016; Rubin et al., 2015).

On the other hand, affinity for solitude can have positive effects on adolescents' social, school, and psychological adjustment by fulfilling the need for autonomy and facilitating its contributions to the attainment of individual achievement. Affinity for solitude allows adolescents to engage in self‐reflection, pursue personal interests, and work on tasks requiring concentration and self‐regulation, and at the same time, improve thoughtful and deliberate decision‐making to reduce risky and deviant behaviors (Thompson et al., 2020; Weinstein et al., 2023). Adolescents who willingly choose solitude are likely to experience autonomy and develop a sense of competence and effectiveness, which may contribute to leadership in peer groups (Mayhew et al., 2007; Nguyen et al., 2022; Thomas, 2023). This argument is consistent with the broad view that positive emotional experience may enhance one's social–cognitive strengths for performance on tasks through the “broaden‐and‐build” processes (e.g., expanding thought‐action repertoires in problem‐solving and improving enduring personal resources, such as intellectual, social, and psychological assets; Fredrickson, 2001). The experience has been shown to foster low‐arousal positive emotions, such as calmness and inner peace, which help adolescents manage stress and cope with challenges and distress (Nguyen et al., 2022).

Given its potential benefits and costs, it seems reasonable to argue that adolescents need to maintain a balanced or moderate level of affinity for solitude to achieve optimal developmental outcomes. Adolescents with affinity for solitude at an appropriate or “just right” level may get most benefits, achieving a healthy balance between social interaction and sense of autonomy (Bowker & Raja, 2011), whereas those with excessive or insufficient such experience may not be able to engage in adequate social interaction for learning from others or self‐directed growth (Nguyen et al., 2022). According to the Confucian principle of *Zhong Yong* (the doctrine of the mean), an indigenous Chinese construct that provides guidance for human social behavior, a balance and moderation should be maintained in all aspects of life, including emotions and social interactions (Yang et al., 2016). Thus, both high and low affinity for solitude may be viewed as socially undesirable as they deviate from the cultural expectation. It should be noted that according to the developmental theories (e.g., Kagitcibasi, 2005; Maccoby & Martin, 1983; Ryan & Deci, 2000), the developmental dynamics of the needs for autonomy and connectedness during adolescence may be universal. Whereas specific cultural values such as *Zhong Yong* may indicate how the balance is expressed and socially evaluated, the benefits of a moderate level of affinity for solitude and maladaptive functions of insufficient and excessive affinity for solitude in adolescents' adjustment are likely to appear across social and cultural settings.

Coplan et al. (2021) argue that a moderate level of solitude may be ideal for adolescents' adjustment as it aligns with their desire for “relaxation and restoration,” and at the same time, avoids potential harms associated with complete social isolation. This perspective focuses on the behavioral aspect of solitude—how much time adolescents actually spend alone. The present study was mainly concerned with the positive emotional aspect of solitude, that is, enjoyment of solitary activities. Given the differences between the behavioral and emotional aspects of solitude (Swets & Cox, 2022), a study of affinity for solitude and its relations with adjustment may help us achieve a new and more comprehensive understanding of the nature and function of adolescents' experiences of solitude. We argue that it is not only the amount of time adolescents spend alone, but also the extent to which they emotionally enjoy solitude that may account for potential curvilinear associations with adjustment.

## THE PRESENT STUDY

The primary purpose of the present 1‐year longitudinal study was to examine the nonlinear associations between affinity for solitude and social, academic, and psychological adjustment in Chinese adolescents. The participants were eighth‐grade students in junior high schools in China, a developmental stage where solitary experiences and affinity for solitude are increasingly common (Danneel et al., 2018; Hu et al., 2022) and crucial for shaping adjustment outcomes (Patton et al., 2016). Mid‐adolescence has been viewed as a particularly important period in the development of constructive solitude functions, such as self‐reflection, autonomy, and emotion regulation (Larson, 1997). During this period, adolescents often engage with solitude more deliberately and use it for constructive purposes, such as managing emotions and fostering personal growth. Affinity for solitude was assessed through adolescents' self‐reports. Data on adjustment and adjustment problems across social (prosociality, leadership, peer preference, aggression, behavioral problems), academic (learning problems), and psychological (school attitudes, loneliness) domains were collected from multiple sources including self‐reports, peer assessments, teacher ratings, and school records. The domains of adjustment were selected to capture how well adolescents adapted to their school environment (Blyth et al., 1983).

According to Daly and Willoughby (2020), it is necessary to control for social anxiety in the study of positive attitudes toward solitude because some adolescents may report enjoying solitary activities due to their anxiety in social situations. Thus, we collected data on social anxiety using self‐reports. Based on the previous discussion, we hypothesized that moderate levels of affinity for solitude would be associated with more positive adjustment and fewer problems in social, academic, and psychological domains relative to high or low levels of affinity for solitude.

We checked gender differences in the relations between affinity for solitude and adjustment. Due to gender stereotypes in society, girls are often expected to be more relationship‐oriented, whereas boys are expected to display greater independence (Bauer, 2015; Cuddy et al., 2015; Han & Li, 2009). As such, affinity for solitude may be more acceptable for boys than for girls. If this is the case, affinity for solitude might be associated with positive adjustment more strongly in boys than in girls. We examined this possibility in the study.

## METHOD

### Participants

Participants in this study were eighth‐grade students (*N* = 2675; 1341 boys) attending middle schools in a region primarily consisting of towns, small cities, and surrounding areas in East China. These students were from 55 classes in five regular public schools. The average age of the students was 14 years and 3 months (SD = 6.54 months). These public schools primarily served students from their respective local geographic regions. Stipulated by the Ministry of Education in China, the structure and organization of Chinese schools are similar. Students typically follow similar schedules of courses and activities and remain in the same class over the years. Each class is overseen by a head teacher who teaches a major course, responsible for the social and daily activities of the class, thereby being familiar with the students. Students are encouraged to engage in various social and academic activities in the school, fostering extensive opportunities for interaction among classmates.

The majority of the participants came from families of low to middle socioeconomic backgrounds. In the sample, 86.63% of the fathers and 93.04% of the mothers had attained an education level of junior high school or below, 8.82% of the fathers and 4.53% of the mothers had a senior high school education, 3.82% of the fathers and 2.09% of the mothers had occupational or technical school training, and 0.73% of the fathers and 0.34% of the mothers had a college or above college education. Most participants were of the Han ethnic nationality, which constitutes over 90% of China's population.

From the original sample, 2250 (84%) students participated in a follow‐up study 1 year later. Multivariate analysis of variance (MANOVA) revealed nonsignificant differences between the students who participated in the follow‐up study and those who did not on the variables at Time 1, Wilks' *λ* = 0.97, *F*(10, 2325) = 0.82, *p* = .61. We conducted an a priori power analysis to determine the sample size. The analysis indicated that a sample size of 92 participants would achieve 80% power to detect a medium effect (*f* ^2^ = 0.15) at a significance level of 0.05 in a multiple regression model with five predictors, including a quadratic term. Furthermore, based on guidelines from Gelman et al. (2020), estimating an interaction effect that is half the size of a main effect requires ~16 times the sample size. Accordingly, our final sample size of 2250 participants provided sufficient power to detect quadratic effects.

### Measures

#### Affinity for solitude

Adolescents' affinity for solitude was assessed using a self‐report measure adapted from the *Child Social Preference Questionnaire* (Coplan et al., 2013). The measure includes four items (“I enjoy being by myself,” “I like doing things alone,” “I like spending time alone in my room,” “I am happy when doing things alone”). Adolescents were asked to respond to the items on a 5‐point scale, ranging from 1 (*never*) to 5 (*always*). The average score of the responses was computed, with higher scores indicating greater enjoyment of solitude. The measure has been used and shown to be reliable and valid in other studies with Chinese children and adolescents (e.g., Chen, Zhou, Li, Liu, & Cui, 2024; Hu et al., 2022). The internal reliability (Cronbach's α) of the measure was 0.85 in the present study.

#### Social anxiety

Adolescents' social anxiety was assessed using the Chinese version of the *Social Anxiety Scale for Children‐Revised* (La Greca & Stone, 1993). Participants responded to 15 items (e.g., “I worry about what other kids think of me,” “I feel nervous when I'm around certain kids”) using a 5‐point scale, ranging from 1 (*not at all true*) to 5 (*always true*). The average score of the items was calculated, with higher scores indicating greater social anxiety. The measure was used and shown to be reliable and valid in previous studies with Chinese adolescents (e.g., Chen, Zhou, Li, Liu, & Cui, 2024; Zhang & Eggum‐Wilkens, 2018). The internal reliability of the measure was 0.90 in the present study.

#### Peer‐assessed prosociality and aggression

Adolescents' prosociality and aggression were assessed using a peer assessment measure adapted from *The Revised Class Play* (Masten et al., 1985). The measure included four items for prosociality (e.g., “Is willing to help others,” “Is polite to others”) and seven items for aggression (e.g., “Picks on other kids,” “Gets into a lot of fights”). For each item, students were asked to nominate up to three classmates who best fit the description. The number of nominations received from all classmates was used to compute item scores for each student. The item scores were calculated by standardizing the nomination scores within the class (z‐scores) to adjust for differences in the number of nominators. The variables of prosociality and aggression were computed by averaging the standardized scores of the corresponding items, with higher scores indicating greater levels of either prosociality or aggression. The measure was used and shown to be reliable and valid in previous studies with Chinese adolescents (e.g., Zhao et al., 2024; Zhou et al., 2024). Internal reliabilities (Cronbach's α) were 0.77 and 0.76 for prosociality and 0.85 and 0.81 for aggression, at Times 1 and 2, respectively, in the present study.

#### Leadership

In Chinese schools, there are various formal student organizations at different levels (e.g., small group, class, and school), which are often hierarchical in nature. Leaders of these organizations, elected by peers and teachers, are usually believed to be good students in social and behavioral aspects. Leadership at a higher level, such as the school level, usually indicates greater competence. Leaders at the school level, for instance, are often responsible for organizing schoolwide student activities and assisting teachers to achieve school social and academic goals, whereas class leaders (e.g., class committee members) typically help with classroom affairs and small‐group leaders engage in coordination with a smaller number of peers within the class. Data on student leadership were collected from school records. Leadership was coded as follows: Students who were small‐group leaders within the class received a score of 1; students who held leadership positions at the class level and at the school level received scores of 2 and 3, respectively; and students who did not hold leadership positions were given a score of 0. Leadership scores were standardized within the classroom to adjust for differences in classroom size and leadership structure. This information has been shown to be a useful indicator of social competence in Chinese students (Chen, Zhou, Li, Liu, Zhang, et al., 2024).

#### Peer preference

Students were asked to nominate up to three classmates they most liked to be with, and three classmates they least liked to be with (positive and negative nominations). As suggested by other researchers (Coie et al., 1995), cross‐gender nominations were allowed. The nominations received from all classmates were summed and then standardized by the classroom for appropriate comparisons. Following the procedure by Coie et al. (1995), an index of peer preference, indicating how well an individual is liked by peers, was formed by subtracting negative nomination scores from positive nomination scores. This sociometric measure was used and shown to be reliable and valid in Chinese adolescents (e.g., Chen, Zhou, Li, Liu, & Cui, 2024; Liu et al., 2015).

#### Teacher‐rated behavioral problems and learning problems

The head teacher was asked to evaluate each participant in the class using a measure adapted from Hightower et al. (1986). The measure comprised seven items for behavioral problems (e.g., “Overly aggressive to peers (fights),” “Disruptive in class”) and six items for learning problems (e.g., “Having difficulties in learning academic subjects,” “Poorly motivated to achieve”). The teachers rated each student on a 5‐point scale, ranging from 1 (*not at all*) to 5 (*very well*), in terms of how well each item described the student. The item scores were standardized within the class to adjust for the teacher's response style. The average score was computed, with higher scores indicating higher levels of behavioral or learning problems. The measure was used and shown to be reliable and valid in previous studies with Chinese adolescents (e.g., Chen, Zhou, Li, Liu, & Cui, 2024; Liu et al., 2015; Zhao et al., 2024). Internal reliabilities were 0.79 and 0.81 for behavioral problems and 0.79 and 0.80 for learning problems, at Times 1 and 2, respectively, in the present study.

#### School attitudes

Students' attitude toward school was assessed with a self‐report measure adapted from Ladd et al. (1997). Adolescents were asked to respond to 11 self‐statements (e.g., “I like school,” “I am happy in the school”) on a 5‐point scale, ranging from 1 (*not at all true*) to 5 (*always true*). The items were averaged to form the school attitude variable, with higher scores indicating a more positive school attitude. This measure has been used and shown to be reliable and valid in Chinese students (Zhang et al., 2017). Internal reliabilities were 0.82 and 0.84 at Times 1 and 2, respectively, in the present study.

#### Loneliness

Students' loneliness was assessed using a self‐report measure (Asher et al., 1984). Students were asked to respond to 16 self‐statements describing loneliness (e.g., “I have nobody to talk to,” “I feel lonely”) using a 5‐point scale, ranging from 1 (*not at all true*) to 5 (*always true*). The average score of the responses was calculated, with higher scores indicating greater loneliness. The measure was used and shown to be reliable and valid in previous studies with Chinese adolescents (Liu et al., 2015). Internal reliabilities were 0.90 and 0.91 at Times 1 and 2, respectively, in the present study.

### Procedure

We group‐administered to the participants self‐report measures of affinity for solitude, social anxiety, school attitudes, and loneliness, peer assessment measures of prosociality and aggression, and a sociometric nomination measure in the classroom setting. Teachers were asked to complete a rating scale for the students. Data on leadership were obtained from school records. The study received approval from the Institutional Review Board. All students in the schools were invited to participate in the study with no exclusions. Active written consent was obtained from the participants and their parents through the school. The participation rate was ~95% at each time. The measures were administered by a team of faculty and graduate students in China. During administration, the research assistants provided detailed explanations of the procedures and the measures. Data on affinity for solitude and social anxiety were collected at Time 1, and data on adjustment measures were collected at both times. Data collection took place near the end of the school year, primarily in June, at each time.

### Data analytic plan

A series of multiple regression models were used to examine the linear and quadratic effects of affinity for solitude on later social, school, and psychological adjustment variables. For each model, the linear and quadratic terms of Time 1 affinity for solitude were included as predictors, and adolescent gender, the corresponding Time 1 adjustment variable, and Time 1 social anxiety were included as control variables. The analysis was performed using R, version 4.3, with the lavaan package (Rosseel, 2012).

## RESULTS

### Descriptive data

The Little's MCAR test (Little, 1988) for the missing data, which ranged from 0% (peer nominations) to 19% (teacher ratings), indicated that the data were missing completely at random, χ^2^(28) = 35.28, *p* = .14. As recommended by other researchers (Graham, 2009), full information maximum likelihood (FIML) estimation was employed to handle the missing data.

In the sample, the scores of affinity for solitude at Time 1 varied from 1 to 5, with an average score of 2.54, a median of 2.50, and a standard deviation of 0.95. A MANOVA revealed significant main effects of gender on the variables, Wilks' *λ* = 0.83, *F*(18, 1536) = 17.88, *p* < .001, 𝜂^2^ = 0.17. Subsequent univariate analyses indicated that, at both times, girls scored higher on prosociality and peer preference and lower on aggression, behavioral problems, and learning problems than boys, and at Time 1, girls had higher scores on affinity for solitude, social anxiety, leadership, and loneliness than boys. The means and standard deviations for both boys and girls and the correlations among the variables are presented in Tables 1 and 2. The magnitudes of the correlations among adjustment variables were weak to moderate, indicating that the measures tapped different but overlapping aspects of social, school, and psychological adjustment. Classroom and school‐level intraclass correlations were less than 0.05 for the variables, indicating no clustering effects of the classroom or school in the present study.

**TABLE 1:** Means and standard deviations of the variables for boys and girls.

	Boys		Girls		*F*‐value
	Mean	SD	Mean	SD	
Time 1					
Affinity for solitude	2.48	0.98	2.60	0.92	10.64**
Social anxiety	2.55	0.73	2.66	0.68	18.70***
Prosociality	−0.11	0.71	0.12	0.83	58.74***
Leadership	−0.06	0.95	0.10	1.04	16.38***
Peer preference	−0.11	1.65	0.13	1.47	15.53***
Aggression	0.28	1.24	−0.28	0.52	238.80***
Behavioral problems	0.29	1.07	−0.28	0.81	214.70***
Learning problems	0.18	1.02	−0.18	0.93	79.49***
School attitudes	3.66	0.64	3.62	0.57	2.24
Loneliness	2.12	0.62	2.17	0.62	5.74*
Time 2					
Prosociality	−0.15	0.91	0.15	1.06	54.23***
Leadership	−0.01	0.97	0.01	1.03	0.34
Peer preference	−0.07	1.58	0.07	1.55	4.25*
Aggression	0.26	1.25	−0.26	0.54	167.60***
Behavioral problems	0.31	1.06	−0.31	0.79	195.90***
Learning problems	0.20	1.06	−0.19	0.89	73.19***
School attitudes	3.65	0.69	3.63	0.60	0.52
Loneliness	2.10	0.66	2.12	0.61	0.94

* *p* < .05.

** *p* < .01.

*** *p* < .001.

**TABLE 2:** Correlations among variables.

	1.	2.	3.	4.	5.	6.	7.	8.	9.	10.
Time 1										
1. Affinity for solitude										
2. Social anxiety	0.25***									
3. Prosociality	−0.01	−0.09**								
4. Leadership	0.01	−0.05	0.31***							
5. Peer preference	−0.01	0.04	0.23***	0.16***						
6. Aggression	−0.11***	−0.05	−0.04	−0.04	−0.37***					
7. Behavioral problems	−0.04	−0.01	−0.14***	−0.16***	−0.21***	0.41***				
8. Learning problems	−0.02	0.04	−0.33***	−0.36***	−0.21***	0.18***	0.59***			
9. School attitudes	−0.21***	−0.21***	0.17***	0.14***	0.07*	−0.04	−0.09***	−0.16***		
10. Loneliness	0.36***	0.31***	−0.23***	−0.12***	−0.18***	−0.03	0.02	0.11***	−0.46***	
Time 2										
11. Prosociality	0.10***	0.05	0.21***	0.22***	0.41***	−0.20***	−0.19***	−0.26***	0.07*	−0.04
12. Leadership	0.03	−0.04	0.18***	0.32***	0.13***	0.00	−0.07	−0.24***	0.10**	−0.08*
13. Peer preference	0.00	0.04	0.17***	0.07*	0.62***	−0.31***	−0.14***	−0.15***	0.07*	−0.15***
14. Aggression	−0.12***	−0.06	−0.02	0.00	−0.28***	0.80***	0.34***	0.15***	−0.01	−0.03
15. Behavioral problems	−0.04	−0.03	−0.08	−0.08*	−0.18***	0.34***	0.40***	0.31***	−0.09**	0.04
16. Learning problems	−0.05	0.03	−0.22***	−0.26***	−0.20***	0.18***	0.30***	0.47***	−0.16***	0.10***
17. School attitudes	−0.17***	−0.20***	0.16***	0.13***	0.07*	0.03	−0.06	−0.15***	0.55***	−0.31***
18. Loneliness	0.26***	0.26***	−0.18***	−0.10***	−0.13***	−0.03	0.04	0.12***	−0.33***	0.57***

	11.	12.	13.	14.	15.	16.	17.
Time 2							
11. Prosociality							
12. Leadership	0.23***						
13. Peer preference	0.49***	0.09**					
14. Aggression	−0.20***	0.00	−0.34***				
15. Behavioral problems	−0.23***	−0.11***	−0.15***	0.36***			
16. Learning problems	−0.27***	−0.25***	−0.16***	0.17***	0.60***		
17. School attitudes	0.11***	0.11***	0.06	0.01	−0.08*	−0.17***	
18. Loneliness	−0.06	−0.08*	−0.15***	−0.02	0.09**	0.14***	−0.53***

* *p* < .05.

** *p* < .01.

*** *p* < .001.

### Longitudinal relations between affinity for solitude and adjustment variables

**FIGURE 1:**
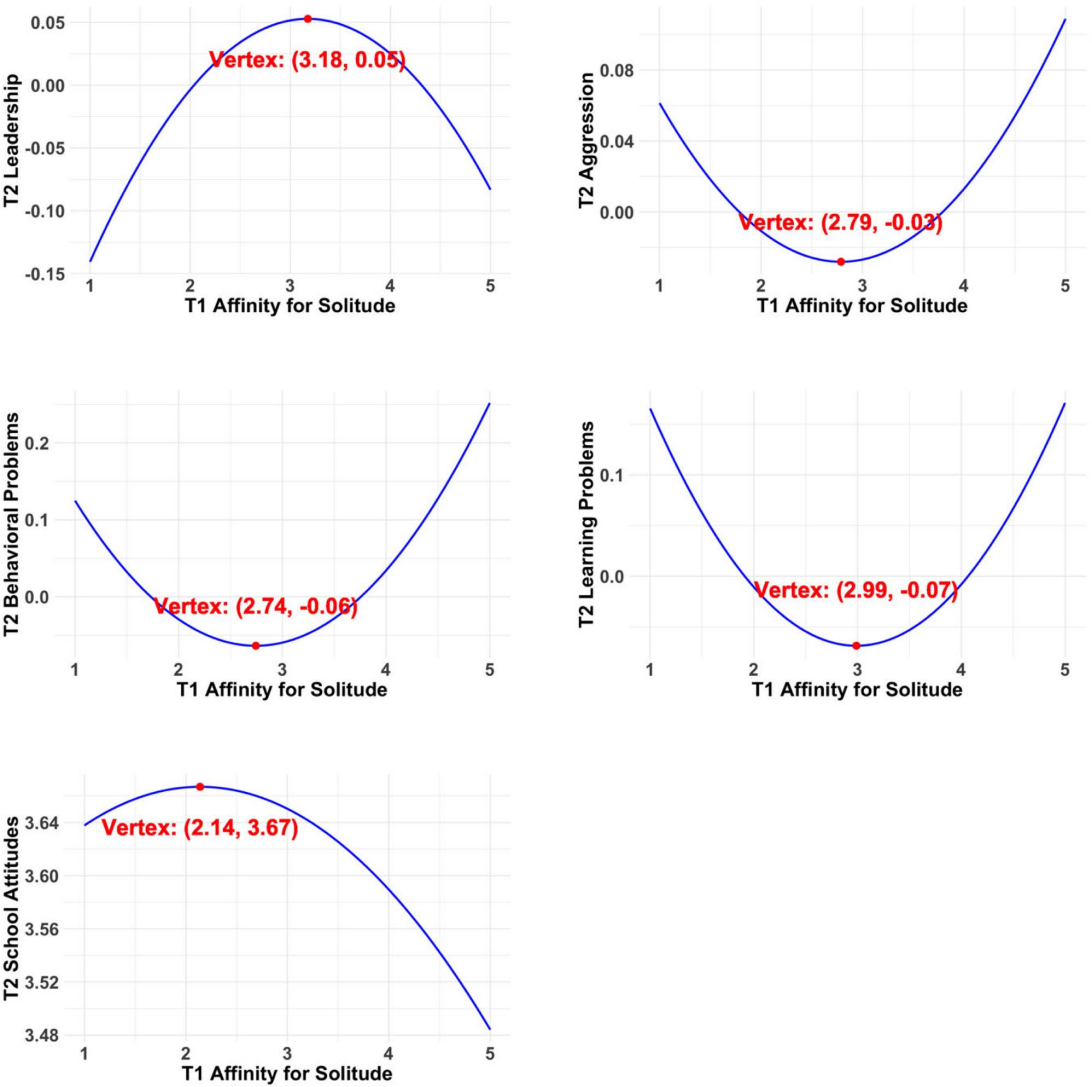
Curvilinear associations between Time 1 affinity for solitude and Time 2 adjustment variables. Gender, Time 1 corresponding adjustment variable, and Time 1 social anxiety was controlled in analyses. The vertex of each curve is highlighted in red.

**FIGURE 2:**
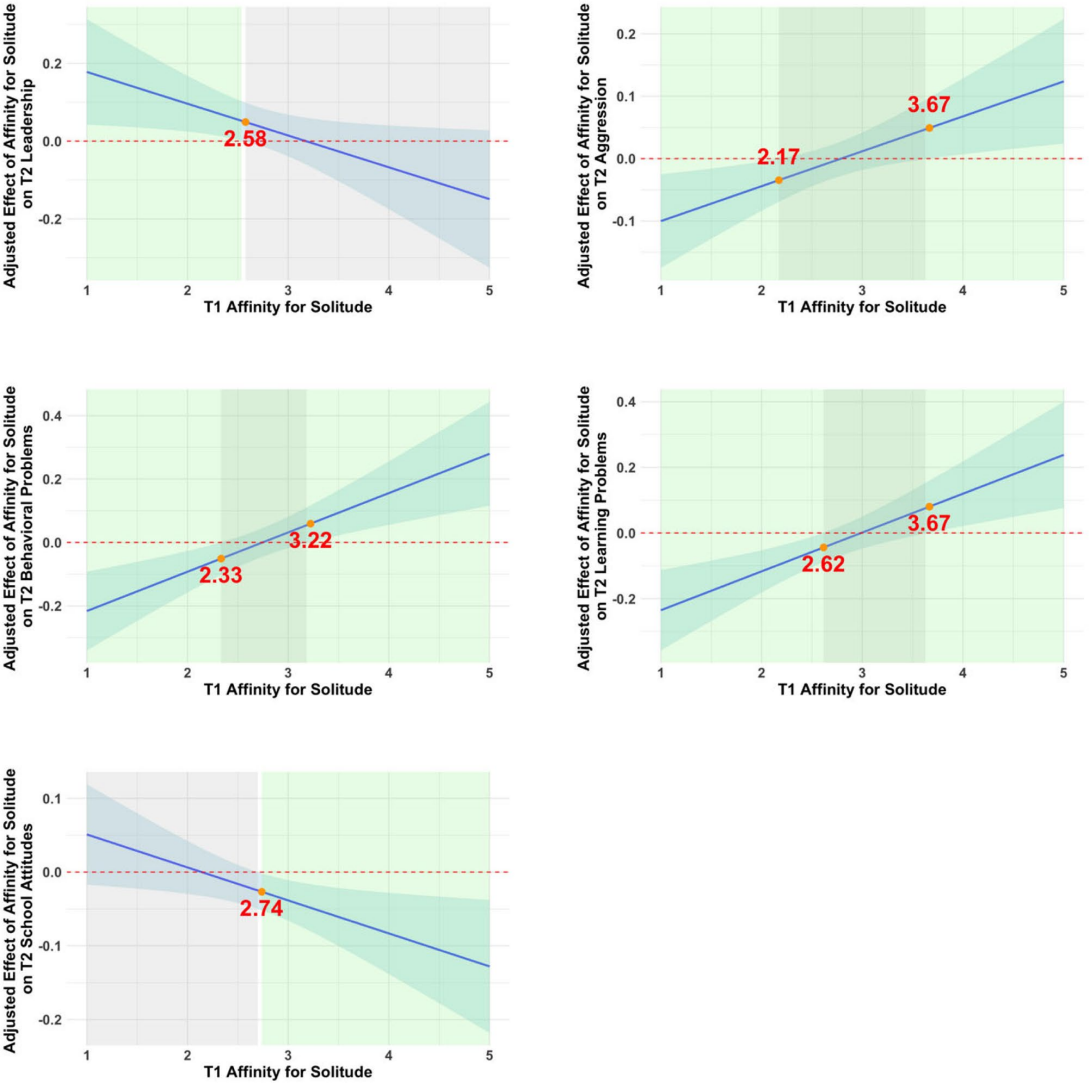
Regions of significance for the adjusted effects of Time 1 affinity for solitude on Time 2 adjustment variables using the Johnson–Neyman technique. The upper and lower bounds of the 95% confidence intervals for the adjusted effects are highlighted. The light green areas represent statistically significant adjusted effects (*p* < .05), while the gray areas indicate nonsignificant effects (*p* > .05). The *x*‐values at the boundaries between significant and nonsignificant regions are marked in red. Gender, Time 1 corresponding adjustment variable, and Time 1 social anxiety was controlled in analyses.

The results of the models, which included both linear and quadratic effects of Time 1 affinity for solitude and control variables in predicting Time 2 adjustment outcomes, are presented in Table 3. The linear term of Time 1 affinity for solitude was positively associated with Time 2 prosociality. The quadratic term of Time 1 affinity for solitude was significantly associated with Time 2 leadership, aggression, behavioral problems, learning problems, and school attitudes. Figures 1 and 2 illustrate the nature of these relations and highlight the regions of significance. As shown in Figure 1, the most positive outcomes, characterized by higher leadership and school attitudes and lower levels of aggression, behavioral problems, and learning problems, emerged within the moderate range of affinity for solitude, with the vertices falling between ~2.14 and 3.18 on the 5‐point scale. The Johnson–Neyman plots in Figure 2 indicated that the positive effects of affinity for solitude on adjustment (e.g., higher leadership, more positive school attitudes, and fewer behavioral and learning problems, greater aggression) were at low to moderate levels of affinity for solitude, and that higher levels of affinity for solitude were associated with less optimal adjustment (e.g., more behavioral and learning problems, greater aggression, and lower leadership). Specifically, the relation between Time 1 affinity for solitude and Time 2 leadership exhibited an inverse U‐shaped pattern, showing a significant positive trend at low to moderate levels of affinity for solitude, but this trend became nonsignificant at higher levels of affinity for solitude (above 2.58 on the 5‐point scale). The relations between Time 1 affinity for solitude and Time 2 aggression, teacher‐rated behavioral problems, and teacher‐rated learning problems showed a U‐shaped pattern. These relations were significant and negative at low to moderate levels of Time 1 affinity for solitude (below 2.17, 2.33, and 2.62, respectively), became nonsignificant at moderate to moderately high levels, and were positive and significant at higher levels of Time 1 affinity for solitude (above 3.67, 3.22, and 3.67, respectively). The relation between Time 1 affinity for solitude and Time 2 school attitudes showed an inverse U‐shaped pattern, being nonsignificant at low to moderate levels of affinity for solitude but exhibiting a negative and significant association at higher levels (above 2.74). In general, the results concerning the quadratic effects suggest that adolescents with a moderate level of affinity for solitude had higher scores on positive adjustment and lower scores on adjustment problems than adolescents with higher and lower levels of affinity for solitude. Time 1 affinity for solitude did not significantly predict Time 2 peer preference or loneliness in linear or quadratic terms. The linear effect of Time 1 affinity for solitude on Time 2 prosociality (Cohen's *f* ^2^ = 0.02) and the quadratic effects on leadership, aggression, behavioral problems, learning problems, and school attitudes were small (*f* ^2^ = 0.002–0.008) but meaningful, consistent with typical effect sizes observed in interaction and quadratic effects in developmental research (Aguinis et al., 2005).

**TABLE 3:** Results of predicting Time 2 adjustment variables based on Time 1 variables.

	*B*	SE	*t* value	95% CI
T2 Prosociality				
Prosociality	0.708	0.014	48.906***	(0.680, 0.736)
Social anxiety	0.017	0.016	1.025	(−0.015, 0.049)
Affinity for solitude (linear)	0.107	0.052	2.076*	(0.006, 0.209)
Affinity for solitude (quadratic)	−0.015	0.009	−1.609	(−0.033, 0.003)
T2 Leadership				
Leadership	0.328	0.023	14.452***	(0.283, 0.372)
Social anxiety	−0.037	0.034	−1.095	(−0.103, 0.029)
Affinity for solitude (linear)	0.259	0.105	2.466*	(0.053, 0.466)
Affinity for solitude (quadratic)	−0.041	0.019	−2.163*	(−0.078, −0.004)
T2 Peer preference				
Peer preference	0.619	0.017	37.003***	(0.586, 0.652)
Social anxiety	0.070	0.037	1.866	(−0.004, 0.143)
Affinity for solitude (linear)	0.102	0.117	0.866	(−0.129, 0.332)
Affinity for solitude (quadratic)	−0.018	0.021	−0.849	(−0.060, 0.024)
T2 Aggression				
Aggression	0.841	0.014	58.561***	(0.813, 0.869)
Social anxiety	−0.028	0.018	−1.533	(−0.065, 0.008)
Affinity for solitude (linear)	−0.156	0.058	−2.670**	(−0.271, −0.041)
Affinity for solitude (quadratic)	0.028	0.011	2.648**	(0.007, 0.049)
T2 Behavioral problems				
Behavioral problems	0.340	0.024	14.309***	(0.293, 0.387)
Social anxiety	−0.020	0.031	−0.660	(−0.080, 0.040)
Affinity for solitude (linear)	−0.340	0.097	−3.519***	(−0.530, −0.151)
Affinity for solitude (quadratic)	0.062	0.017	3.556***	(0.028, 0.096)
T2 Learning problems				
Learning problems	0.455	0.023	20.232***	(0.411, 0.499)
Social anxiety	0.036	0.030	1.174	(−0.024, 0.095)
Affinity for solitude (linear)	−0.354	0.095	−3.710***	(−0.541, −0.167)
Affinity for solitude (quadratic)	0.059	0.017	3.441***	(0.025, 0.093)
T2 School attitudes				
School attitudes	0.565	0.020	28.349***	(0.526, 0.604)
Social anxiety	−0.070	0.017	−4.124***	(−0.104, −0.037)
Affinity for solitude (linear)	0.096	0.053	1.807	(−0.008, 0.200)
Affinity for solitude (quadratic)	−0.022	0.010	−2.338*	(−0.041, −0.004)
T2 Loneliness				
Loneliness	0.555	0.020	27.507***	(0.515, 0.594)
Social anxiety	0.068	0.017	4.049***	(0.035, 0.100)
Affinity for solitude (linear)	0.010	0.051	0.187	(−0.091, 0.110)
Affinity for solitude (quadratic)	0.004	0.009	0.453	(−0.014, 0.022)

*Note*: Gender was controlled in the analyses.

* *p*< .05.

** *p* < .01.

*** *p* < .001.

We tested gender differences using multigroup analysis by comparing models with all paths constrained to be equal across gender with the unconstrained models. There were no significant gender differences, Δ𝜒^2^ (4) = 0.94, *p* = .919, to Δ𝜒^2^ (4) = 3.38, *p* = .500, indicating that the results were consistent for both boys and girls. We also examined social anxiety as a potential moderator by including the interaction terms between the predictors and social anxiety in the models. These interaction effects were not significant, suggesting that the associations did not vary by levels of social anxiety. Therefore, the interaction terms were not included in the final models.

We conducted a path analysis including all outcome variables simultaneously. The omnibus model did not achieve satisfactory fit. Nevertheless, the patterns of the results were consistent with those from the separate models, supporting the robustness of the findings. Multiple‐group comparisons showed no significant gender differences. The results are presented in Table S2.

## DISCUSSION

Affinity for solitude becomes increasingly prevalent since early adolescence (Coplan et al., 2019; Daly & Willoughby, 2020; Danneel et al., 2018). The present study showed a positive linear relation between affinity for solitude and later prosociality. Moreover, the study revealed a curvilinear pattern of the relations between affinity for solitude and multiple adjustment outcomes, suggesting that a moderate level of affinity for solitude was more beneficial than high and low levels of affinity for solitude for adolescents' adjustment. The results may help us understand and potentially reconcile the inconsistent results of the previous studies (e.g., Borg & Willoughby, 2022; Chen, Zhou, Li, Liu, & Cui, 2024). The findings provide valuable information about the implications of under‐, over‐, and adequate affinity for solitude for adolescent development in social, school, and psychological domains.

### Adolescents' affinity for solitude and social, school, and psychological adjustment

The study first indicated a positive linear association between affinity for solitude and prosociality, suggesting that adolescents who enjoy solitude tend to exhibit greater prosocial behavior. This aligns with prior research indicating that prosocial behavior often requires self‐regulatory capacity to manage conflicts between self‐oriented and other‐oriented motivations (DeWall et al., 2008). Affinity for solitude is believed to help regulate affective states, promoting the display of prosocial behavior (Nguyen et al., 2018).

Affinity for solitude at low to moderate levels, but was not at high levels, was significantly and positively associated with leadership. Increasing affinity for solitude from low to moderate levels seems to help adolescents' leadership development by fostering essential power‐related traits such as independence (Magee & Smith, 2013; Manokara et al., 2024). Research has shown that independence and social distance are positively associated with power in the group (Magee & Smith, 2013), particularly when decision‐making is required to achieve group goals (Manokara et al., 2024). The results also showed that a higher‐than‐moderate level of affinity for solitude did not confer additional benefits for leadership, with the positive effects plateauing at a moderate level. The results suggest that moderate affinity for solitude may be sufficient to maintain leadership.

Significant quadratic associations emerged between affinity for solitude and aggression, behavioral problems, and learning problems, indicating the risks associated with both high and low levels in adolescents' social and school adjustment. Largely consistent with these patterns, affinity for solitude at higher levels, but not at low to moderate levels, was significantly and negatively associated with school attitudes. Adolescents with low affinity for solitude may be inclined to engage in a variety of social activities, particularly informal and unstructured peer activities, which may serve as a context for exposure to interpersonal issues and for displaying deviant behaviors (Anderson & Hughes, 2009; Chen & Chen, 2019; Tomova et al., 2021). Adolescents with low affinity for solitude may also miss opportunities to leverage calmness and restoration in solitude to cope with daily stressors or escape peer pressure and social demands (Nguyen et al., 2018; Weinstein et al., 2023). Furthermore, low affinity for solitude may interfere with self‐directed learning and weaken the ability to work independently and concentrate on tasks (Thomas, 2023).

On the other hand, high affinity for solitude may prevent adolescents from adequate social interaction and thus deprive them of opportunities to learn social standards and appropriate behaviors. In the collectivistic Chinese context where participation in group activities and contributions to group well‐being are valued, adolescents with high affinity for solitude may face challenges integrating into the environment. The social disapproval that these adolescents experience may trigger frustration, anger, and other negative emotional reactions, which, in turn, may result in behavioral problems (Elmore & Huebner, 2010; Swets & Cox, 2022). High affinity for solitude may also create obstacles in obtaining assistance and group collaboration on academic tasks and exacerbate learning difficulties. At the same time, the experiences may reinforce adolescents' negative school attitudes, such as disengagement or dissatisfaction with the school environment.

The lack of significant differences in school attitudes at low to moderate levels of affinity for solitude suggests that the pattern of the relation may be different from those for the behavioral or social outcomes (e.g., externalizing and learning problems). The behavioral and social variables were assessed using teacher or peer reports, which might be more reflective of social evaluations based on the standards and expectations in school and thus more sensitive to individual differences in affinity for solitude. Self‐reported school attitudes, on the other hand, indicate adolescents' perceptions and feelings about school life. The formation of and change in school attitudes may involve more complicated social–cognitive processes based on repeated experiences in the school environment and seem to be relatively robust to variations in affinity for solitude at low to moderate levels. It is also possible that Chinese schools emphasize and promote group participation and peer interaction in most activities, which may fulfill the social interaction needs of adolescents with low to moderate affinity for solitude. However, students with high affinity for solitude may experience an evident misfit between their desire for autonomy and the requirements of the collectivistic, teacher‐led school environment, which may contribute to more negative school attitudes. Of course, further research is clearly needed on this issue. Taken together, our results suggest that in general, a moderate level of affinity for solitude appears to be optimal for supporting balanced social engagement, self‐regulated functioning, and academic performance. In contrast, low or high levels of affinity for solitude may impede adolescents' social‐behavioral and school adjustment.

It should be noted that the quadratic effects were small. Such magnitudes are typical for interaction and nonlinear terms in developmental research and are meaningful (Aguinis et al., 2005). In this study, the standard errors associated with the quadratic coefficients were very small, indicating high precision in the estimates, which resulted in statistically detectable nonlinear patterns. Small curvatures in the affinity for solitude–adjustment associations represented substantial proportions of adolescents (~17% of adolescents scored one standard deviation below the mean, ~16% of the adolescents scored one standard deviation above the mean of affinity for solitude in the data), suggesting that nonlinearity captured a sizable segment of the participants. In other words, small changes in the slope at low versus high levels of affinity for solitude may indicate relevant distinct patterns of its associations with social and psychological outcomes. Practically, small quadratic effects, reflecting cumulative developmental processes, may also be important, particularly for applied work with large populations (Carey et al., 2023). In this study, the quadratic patterns showed different outcomes for adolescents with low, moderate, and high affinity for solitude, which would be obscured in the linear models. The information about the potential benefits for healthy adjustment and risks for developing problems of affinity for solitude at different levels is valuable for intervention and education.

The results indicated that adolescents' affinity for solitude did not significantly contribute to peer preference or loneliness. The nonsignificant contribution of affinity for solitude to peer preference was consistent with the previous results of cross‐sectional studies (e.g., Chen, Zhou, Li, Liu, & Cui, 2024), suggesting that affinity for solitude may not elicit evident reactions from peers. It is possible that peers understand the motivations for and experiences of solitary activities and thus respect the action of adolescents who display affinity for solitude. The results also suggest that, as argued by Borg and Willoughby (2022), affinity for solitude and loneliness represent distinct constructs; whereas the former indicates a positive emotional inclination toward spending time alone, the latter indicates social dissatisfaction due to a gap between desired and actual social relationships (Asher & Paquette, 2003). Adolescents with a high affinity for solitude do not necessarily develop increased feelings of loneliness, presumably because their affinity for solitude is self‐determined and fulfilling based on their own choice.

## LIMITATIONS AND FUTURE DIRECTIONS

Several limitations and weaknesses in the study should be noted. First, based on the literature (e.g., Coplan et al., 2021; Weinstein et al., 2023), we discussed the results, particularly those concerning the quadratic associations between affinity for solitude and adjustment in terms of social‐relational and social‐cognitive processes, such as balanced social interaction and pursuit of self‐interest. However, these processes were not assessed in this study. Future studies using various methods, such as behavioral observations and interviews, may help better understand how adolescents with different levels of affinity for solitude engage with peers in diverse classroom contexts and the motivations.

Second, this study was conducted with adolescents in junior high school. One needs to be careful to generalize the results to other developmental periods, such as early childhood or senior high school. Research has shown that young adolescents are likely to experience solitude more negatively, whereas older adolescents and emerging adults tend to hold more positive views of solitude as a source of restoration and autonomy (Cheng et al., 2025; Coplan et al., 2019). Thus, the “optimal” level of affinity for solitude for adjustment may vary across developmental periods during adolescence and emerging adulthood. It will be important to investigate this issue in the future.

Third, this study focused on the role of affinity for solitude in contributing to later adjustment. We did not examine the antecedents of affinity for solitude. It will be interesting in future research to explore the social and individual factors that predict affinity for solitude.

Fourth, while girls scored higher on affinity for solitude than boys, no significant gender differences were found in the associations between affinity for solitude and adjustment, suggesting the associations were consistent for boys and girls. Given the argument about gender‐related socialization norms and expectations about adolescents' behaviors (e.g., Cuddy et al., 2015; Han & Li, 2009), this issue should be further examined in the future.

Fifth, the measure of affinity for solitude used in the study focused on the general emotional experience of enjoyment toward solitude without specifying the *context* in which solitude occurred (e.g., spending time alone at home or school). It is possible that affinity for solitude may vary across contexts with potentially different implications for adjustment. Researchers should use context‐specific measures in future research to help us further understand adolescents' affinity for solitude.

Sixth, as another limitation, multiple tests were conducted in the study, which might increase Type I errors. The consistent results from the omnibus model including all outcome variables simultaneously and the separate models supported the robustness of the findings. Nevertheless, our discussion focused mainly on the general pattern of the results. The interpretation of specific findings should be made with caution.

Finally, this study was conducted in a region consisting mainly of towns, small cities, and surrounding areas in East China. Substantial regional differences exist in China in social and economic development. Thus, generalization of the results of this study should be made with caution to other regions, such as larger urban centers where self‐oriented values may be more salient or more remote rural areas where traditional values are more maintained. Relatedly, the sample in the present study was relatively socioeconomically homogeneous, with most parents reporting lower levels of formal education. This homogeneity may constrain the generalizability of the findings. The relatively limited educational and social resources in the families might not provide adolescents with adequate access to private space at home, extracurricular opportunities for self‐identity exploration, and materials (e.g., books) that support constructive solitude experiences. The socioeconomic and educational conditions may affect adolescents' experiences of solitude and their relations with adjustment. It will be important to conduct research on affinity for solitude and adjustment in more socioeconomically diverse samples.

Despite the limitations, the results of this study constitute a significant contribution to our understanding about affinity for solitude in adolescents. The study has practical implications for parents, teachers, and professionals. For example, our findings indicate that a moderate level of affinity for solitude (~2.14–3.18 on the 5‐point scale ranging from 1 to 5, with 1 indicating never and 5 indicating always) is optimal for adolescents' social, school, and psychological adjustment. Thus, it is important to encourage adolescents to maintain a balance between a desire to spend time alone and engaging in social interaction. The moderate range is substantial, spanning from below‐average to above‐average levels with the sample mean of 2.54 [SD = 0.95]. (Note that the estimated vertices differed across outcomes (2.14–3.18), which was likely due to measurement‐related factors, such as differences in the scales and distributions.) The relatively wide range of optimal affinity for solitude suggests that adolescents can benefit from different degrees of enjoyment of solitude within the range. For adolescents with low affinity for solitude beyond the range, it may be useful to help them develop independent skills and acquire enjoyable experiences with constructive solitary activities. For adolescents with high affinity for solitude, on the other hand, parents and teachers should provide support and guidance for them to participate in group activities that align with their interests.

## AUTHOR CONTRIBUTIONS

All listed authors have contributed to the manuscript substantially and have agreed to the final submitted version. Jiaxi Zhou served as lead for conceptualization, formal analysis, writing original draft, and writing—review and editing. Xinyin Chen served as lead for conceptualization, methodology, supervision, and writing—review and editing. Dan Li served as lead for conceptualization, methodology, resources, and supervision, project administration, and writing—review. Junsheng Liu served as lead for conceptualization, methodology, resources, and supervision, project administration, and writing—review. Tong Zhou served as lead for investigation and project administration.

## CONFLICT OF INTEREST STATEMENT

The authors report no conflict of interest.

## ETHICS STATEMENT

This research complied with the APA's ethical standards. The ethical approval was obtained from the Institutional Review Board of Shanghai Normal University.

## PATIENT CONSENT STATEMENT

Parent/guardian consent was obtained.

## Supporting information


**Table S1.** Distinctions between affinity for solitude and related constructs.
**Table S2.** Results of the single path model predicting Time 2 adjustment variables from Time 1 affinity for solitude.

## Data Availability

The datasets generated and/or analyzed during the current study are not publicly available but are available from the corresponding author on reasonable request.
