# Supplementary material for: Linear and curvilinear associations between affinity for solitude and adjustment in adolescence
Source: J Res Adolesc. 2025 Dec 29;36(1):e70129. doi: 10.1111/jora.70129 (PMC12746541; doi:10.1111/jora.70129)
Supplement: Supplementary file 1 — Table S1. Distinctions between affinity for solitude and related constructs. Table S2. Results of the single path model predicting Time 2 adjustment variables from Time 1 affinity for solitude. [file JORA-36-0-s001.docx]

Linear and Curvilinear Associations Between Affinity for Solitude and Adjustment in Adolescence

**Supplementary Materials**

Table S1

*Distinctions Between Affinity for Solitude and Related Constructs*

| Construct | Definition | Primary Motivation | Emotional Experience with Solitude |
| --- | --- | --- | --- |
| Affinity for solitude | Enjoyment of solitary activities or positive emotional experience with spending time alone | Intrinsic enjoyment of solitude | Positive affect is essential |
| Preference for solitude | Tendency to choose solitary activities over social interactions | Low interest in interacting with others | Emotional valence varies (neutral/positive/negative) |
| Unsociability | Low motivation for social engagement despite low social anxiety | Indifference to social activities | Typically neutral |
| Introversion | Personality trait to focus on the internal world and perceive low reward value of social interaction | Dispositional orientation | Emotional valence varies |

Table S2

*Results of the Single Path Model Predicting Time 2 Adjustment Variables from Time 1 Affinity for Solitude*

|  | *B* | *SE* | *t value* | *95% CI* |
| --- | --- | --- | --- | --- |
| ***T2 Prosociality*** |  |  |  |  |
| Prosociality | .669 | .014 | 47.84*** | (.642, .696) |
| Social Anxiety | .017 | .016 | 1.04 | (-.015, .049) |
| Affinity for Solitude (Linear) | .111 | .052 | 2.15* | (.009, .213) |
| Affinity for Solitude (Quadratic) | -.015 | .009 | -1.64 | (-.033, .003) |
| ***T2 Leadership*** |  |  |  |  |
| Leadership | .302 | .022 | 13.51*** | (.259, .345) |
| Social Anxiety | -.047 | .033 | -1.44 | (-.112, .018) |
| Affinity for Solitude (Linear) | .280 | .103 | 2.73** | (.078, .482) |
| Affinity for Solitude (Quadratic) | -.044 | .018 | -2.41* | (-.079, -.009) |
| ***T2 Peer Preference*** |  |  |  |  |
| Peer Preference | .585 | .016 | 37.21*** | (.554, .616) |
| Social Anxiety | .070 | .037 | 1.89 | (-.003, .143) |
| Affinity for Solitude (Linear) | .115 | .117 | 0.98 | (-.114, .344) |
| Affinity for Solitude (Quadratic) | -.021 | .021 | -0.98 | (-.062, .020) |
| ***T2 Aggression*** |  |  |  |  |
| Aggression | .813 | .014 | 56.62*** | (.785, .841) |
| Social Anxiety | -.028 | .018 | -1.52 | (-.063, .007) |
| Affinity for Solitude (Linear) | -.166 | .058 | -2.84** | (-.280, -.052) |
| Affinity for Solitude (Quadratic) | .029 | .011 | 2.77** | (.008, .050) |
| ***T2 Behavioral Problems*** |  |  |  |  |
| Behavioral Problems | .308 | .020 | 15.25*** | (.268, .348) |
| Social Anxiety | -.009 | .029 | -0.32 | (-.066, .048) |
| Affinity for Solitude (Linear) | -.349 | .093 | -3.77*** | (-.531, -.167) |
| Affinity for Solitude (Quadratic) | .063 | .017 | 3.74*** | (.030, .096) |
| ***T2 Learning Problems*** |  |  |  |  |
| Learning Problems | .387 | .019 | 20.02*** | (.350, .424) |
| Social Anxiety | -.009 | .029 | -0.32 | (-.066, .048) |
| Affinity for Solitude (Linear) | -.354 | .095 | -3.71*** | (-.540, -.168) |
| Affinity for Solitude (Quadratic) | .041 | .011 | 3.44*** | (.019, .063) |
| ***T2 School Attitudes*** |  |  |  |  |
| School Attitudes | .522 | .018 | 29.00*** | (.487, .557) |
| Social Anxiety | -.078 | .017 | -4.60*** | (-.112, -.044) |
| Affinity for Solitude (Linear) | .093 | .053 | 1.76 | (-.011, .197) |
| Affinity for Solitude (Quadratic) | -.023 | .010 | -2.36* | (-.043, -.004) |
| ***T2 Loneliness*** |  |  |  |  |
| Loneliness | .519 | .018 | 28.61*** | (.484, .554) |
| Social Anxiety | .076 | .017 | 4.56*** | (.043, .109) |
| Affinity for Solitude (Linear) | .015 | .051 | 0.29 | (-.085, .115) |
| Affinity for Solitude (Quadratic) | .004 | .009 | 0.48 | (-.014, .022) |

*Note.* Gender was controlled in the analyses. * *p* < .05. ** *p* < .01. *** *p* < .001.

We also conducted multiple-group comparisons to test gender differences in the single-path model by comparing models with all paths constrained to be equal across gender with the unconstrained models. The results were nonsignificant Δ𝜒^2^ (32) = 32.714, *p* = .432, indicating that the associations between affinity for solitude and adjustment did not differ by gender.
